# Examining the Impact of Socioeconomic Factors and Lifestyle Habits on Obesity Prevalence Among Male and Female Adolescent Students in Asser, Saudi Arabia

**DOI:** 10.7759/cureus.43918

**Published:** 2023-08-22

**Authors:** Ayoub A Alshaikh, Abdulrahman S Alqahtani, Fahad A A AlShehri, Abdulrahman M Al Hadi, Meshal Mohammed M Alqahtani, Omair M Alshahrani, Meteb A Albraik, Saad A Alamri, Ramy M Ghazy

**Affiliations:** 1 Family & Community Medicine Department, King Khalid University, Abha, SAU; 2 Medical School, King Khalid University, Abha, SAU; 3 Medicine and Surgery Department, King Khalid University, Abha, SAU; 4 College of Medicine, King Khalid University, Abha, SAU; 5 Abha Health Sector, General Directorate of Health Affairs, Abha, SAU; 6 Public Health Department, General Directorate of Health Affairs, Aseer Region, Abha, SAU; 7 Tropic Health Department, High Institute of Public Health - Alexandria University, Alexandria, EGY

**Keywords:** saudi arabia, body mass index (bmi), stress, sedentary lifestyle, cigarette smoking, obesity

## Abstract

Background

Understanding the relationships between obesity and lifestyle factors is essential for the effective prevention and management of obesity in youth. This study aimed to investigate the association between sociodemographic factors, lifestyle elements such as physical activity and social stress, and the prevalence of overweight and obesity among Saudi adolescents in the Aseer region.

Methodology

From December 2022 to March 2023, we conducted a cross-sectional study using the multi-stage stratified random sampling technique. The study included Saudi male and female adolescents aged 12-19 years attending middle and high schools. Ordinal logistic regression was used to analyze the association between the ordinal dependent variable, classified into weight groups (normal, overweight, obese), and the independent variables.

Results

Of the total of 512 individuals, 90.4% were aged ≥18 years, 77.5% were males, and 76.8% were urban residents. Of the studied population, 33.6% were overweight, and 20.5% were obese. The prevalence of obesity and overweight was significantly higher among males compared to females (20.9% vs. 19.1% and 36.5% vs. 23.5%, respectively). Multivariate analysis revealed the following factors to be associated with obesity and overweight: female gender (2.31, 95% CI = 1.45-3.71), age 12-17 years (0.53, 95% CI = 0.28-0.97), place of delivery (Tanoma) (2.32, 95% CI = 1.13-4.75), family size of over eight members (0.43, 95% CI = 0.24-0.74), family monthly income of over 20,000 SAR (3.79, 95% CI = 1.38-11.35), being smokers (0.26, 95% CI = 1.31-2.93), experiencing social stress (1.96, 95% CI = 1.96-2.93), engagement in physical activity less than three times a week (0.49, 95% CI = 0.32-0.75), and engagement in physical activity more than three times a week (0.36, 95% CI = 0.22-0.58).

Conclusions

These findings emphasize the importance of addressing demographic, socioeconomic, and lifestyle factors in combating childhood and adolescent obesity through targeted interventions.

## Introduction

The world is currently experiencing a significant epidemiological and nutritional transition, marked by the persistence of nutritional deficiencies such as stunting, anemia, and deficiencies in essential minerals such as iron and zinc. At the same time, there is a concerning increase in the prevalence of obesity, diabetes, and other chronic diseases (non-communicable diseases), including cardiovascular diseases and certain types of cancer. Obesity, in particular, has reached epidemic proportions in developed countries. It is noteworthy that the increase in obesity is not confined solely to developed nations but also extends to developing countries [1]. Furthermore, the increasing prevalence of childhood obesity in developing countries is expected to impose a substantial public health burden [2], as it is associated with various comorbidities [3]. In 2019, the age-standardized disability-adjusted life years (DALYs) associated with malnutrition was 680 (95% uncertainty interval = 507-895) per 100,000 individuals. From 2000 to 2019, the DALY rates exhibited a decline at an annual rate of -2.86%, and they are projected to decrease by 8.4% between 2020 and 2030. The regions with the highest obesity-related DALYs were Eastern Mediterranean and countries with middle sociodemographic index levels [4].

The determination of excess obesity or overweight/obesity in children and adolescents remains a topic of debate, with no universally agreed-upon cut-off points. Williams et al. [5] conducted a study among 3,320 children aged 5-18 years and classified them as obese if the percentage of body fat exceeded 25% for males and 30% for females. On the other hand, the Centers for Disease Control and Prevention defines overweight as at or above the 95th percentile of body mass index (BMI) for age, and at risk for overweight as falling between the 85th and 95th percentiles of BMI for age [6].

The dangers of obesity on health status, quality of life, and life expectancy are garnering increasing awareness [7], and are considered a significant public health concern in the Eastern Mediterranean region [8]. Obesity is a complex disease influenced by genetic, demographic, and lifestyle factors [7,9]. While genetic and demographic variables are non-modifiable, lifestyle factors can be addressed. Studies have revealed that childhood obesity is associated with various lifestyle factors, including sedentary behaviors, physical inactivity, and unhealthy dietary choices [10-12]. However, some studies have not shown consistent associations between childhood obesity and certain lifestyle factors, such as soda consumption or computer use [13]. Obese children face an increased risk of developing type 2 diabetes and early cardiovascular diseases, and obesity during adolescence tends to persist into adulthood [14,15]. Given the long-term consequences of childhood obesity, early identification of elevated BMI and prevention of excessive weight gain are crucial [9].

In Saudi Arabia, where rapid urbanization and nutritional changes have occurred, longitudinal studies have confirmed a steady increase in obesity rates among children and adolescents over the past two decades. A study from the northern region revealed that 12% of male children were overweight, and 7% were classified as obese [16]. Another study from the Riyadh region showed that 13.8% of adolescent males were overweight, while 20.5% were classified as obese [17]. On the other hand, in the southern region of Saudi Arabia, specifically in Abha, the prevalence of overweight adolescent students was much lower at 5%, with 11% of students classified as obese [18].

Information on lifestyle factors associated with obesity in Saudi Arabian adolescents revealed a correlation between unhealthy dietary choices, inactivity, and BMI [17-20]. Therefore, understanding the relationships between obesity and lifestyle factors is essential for the effective prevention and management of obesity in adolescents. We hypothesize that there is a significant association between different lifestyles and obesity among adolescents. It is worth noting that the most recent study undertaken in the Asser region was conducted in 2008 solely involving male participants [18]. Therefore, this study was conducted to bridge this gap and provide a comprehensive understanding of the current situation in the region, considering both genders. In this study, we aimed to investigate the association between sociodemographic factors, lifestyle elements such as physical activity and social stress, and the prevalence of overweight and obesity among Saudi adolescents in the Aseer region.

## Materials and methods

The Aseer region is divided into 16 governorates, namely, Abha, Muhayil, An-Namas, Billasmar, Billahmar, Balqarn, Bareq, Bishah, Khamis Mushayt, Rijal Alma, Tathlith, Sarat Ubaidah, Ahad Rifaydah, Al-Majardah, and Al-Harajah. This cross-sectional survey study included Saudi male and female adolescents aged 12-19 years attending middle and high schools. We excluded adolescents with chronic diseases such as diabetes, heart disease, kidney disease, and anemia. The minimum sample size was determined to achieve a 95% confidence level with a margin of error of ±0.05. Assuming a population proportion with obesity or overweight of 0.50, the minimum sample size was 384. To account for a potential non-response rate of 30%, the sample size was increased to 500.

The sample selection utilized a multistage stratified random sampling technique. In the first stage, stratification was based on cities, and four cities were randomly selected (Abha, Kamis Mushayt, Tanoma, and An-Namas). Then, one school per city (two for males and two for females) was randomly selected. In the second stage, classes from each grade (10, 11, and 12) were selected using simple random sampling. All students in the selected classes were invited to participate in the study. We started the study by disseminating flyers among students that contained the written informed content, which was then signed by their parents or guardians.

We collected data on socioeconomic status (age, sex, residence, place of delivery, living conditions, parental education, and income) and lifestyle habits such as smoking (current smoker, ex-smoker, non-smoker), physical activity (less than three times per week, more than three times per week, no physical exercise), social stress, watching television (TV), and sleeping hours. Trained dieticians and technicians assisted students in answering the questionnaire.

During the study, participants’ body weight was measured using an electronic scale, and height was measured using a stadiometer. Height was measured using a calibrated portable measuring rod to the nearest centimeter while subjects stood in their full standing position without shoes. During weight measurement, participants wore light clothing and were barefooted. Anthropometric measurements were made in the morning by trained researchers using standardized procedures. The variables included body weight and height. To calculate the BMI, the body weight in kilograms was divided by the height squared in meters. To determine overweight and obesity in adolescents aged 15-17 years, we used reference values for BMI specific to the age and sex according to the International Obesity Task Force [21]. For participants aged 18 years or above, the cut-off points for adults were used, defining overweight as 25-29.9 kg/m^2^ and obesity as ≥30 kg/m^2^.

The statistical analysis for this study was performed using SPSS version 27 (IBM Corp., Armonk, NY, USA). Categorical variables were summarized as counts and percentages. To assess significant associations between two independent categorical variables, Pearson’s chi-square test was used. Ordinal logistic regression was used to examine the associations between the ordinal dependent variable, weight categories (normal, overweight, obese), and one or more independent variables. The predictors were age categorized into two groups (12-17 years and ≥18 years), gender (female and male), place of delivery (Abha, Kamis Mushayt, Tanoma, and An-Namas), living condition, family size (below six members, between six and eight members, and above eight members), family income per month (less than 5,000 SAR, 5,000 to 10,000 SAR, 10,000 to 20,000 SAR, and above 20,000 SAR), mother’s education ranging from illiterate to university education, father’s education ranging from illiterate to university education, smoking (ex-smoker, non-smoker, and smoker), social stress (yes or no), watching TV (yes or no), and physical activity (less than three times per week, more than three times per week, no physical exercise). A p-value of less than 0.05 was considered statistically significant.

The study objectives were communicated to parents, and written consent forms were obtained before the study commenced. Parents signed the form for children aged less than 18 years. Ethical approval was obtained from the Research Ethics Committee at King Khalid University (approval number: ECM#2023-403). The study followed the principles of the Helsinki Declaration.

## Results

Of the 512 individuals, the majority of the participants (463, 90.4%) were 18 years or above, with a higher predominance of men (397, 77.5%). Almost three-fourths were living in urban areas (393, 76.8%). Most respondents lived with their families (466, 91.0%) and were non-smokers (399, 77.9%). The educational levels of mothers ranged from illiterate (61, 11.9%) to postgraduate (34, 6.6%), while the educational levels of fathers ranged from illiterate (15, 2.9%) to postgraduate (58, 11.3%). The distribution of family size was as follows: below six members (156, 30.5%), six to eight members (270, 52.7%), and above eight members (86, 16.8%). Concerning monthly income, 22 (4.3%) individuals had an income of less than 5,000 SAR, 86 (16.8%) had an income of 5,000-10,000 SAR, 241 (47.1%) had an income of 10,000-20,000 SAR, and 163 (31.8%) had an income above 20,000 SAR (Table 1).

**Table 1:** Different sociodemographic characteristics of the surveyed adolescents in the Aseer region, Saudi Arabia (n = 512). SAR = Saudi Arabian Riyal

Variable (n = 512)	Demographic characteristics	Number	Percentage
Age	12–17 years	49	9.6
	≥18 years	463	90.4
Sex	Female	115	22.5
	Male	397	77.5
Residence	Rural	119	23.2
	Urban	393	76.8
Living condition	Alone	46	9.0
	Family	466	91.0
Place of delivery	Abha	213	41.6
	Kamis Mushayt	81	15.8
	Tanoma	40	7.8
	An-Namas	178	34.8
Smoking (adolescent)	Ex-smoker	33	6.4
	Non-smoker	399	77.9
	Smoker	80	15.6
Mother’s education	Illiterate	61	11.9
	Primary education	53	10.4
	Preparatory	33	6.4
	Secondary	103	20.1
	University	228	44.5
	Postgraduate	34	6.6
Father’s education	Illiterate	15	2.9
	Primary	42	8.2
	Preparatory	40	7.8
	Secondary	115	22.5
	University	242	47.3
	Postgraduate	58	11.3
Family size	Below 6 members	156	30.5
	6–8 members	270	52.7
	Above 8 members	86	16.8
Income	Less than 5,000 SAR	22	4.3
	5,000–10,000 SAR	86	16.8
	10,000–20,000 SAR	241	47.1
	Above 20,000 SAR	163	31.8

**Figure 1:**
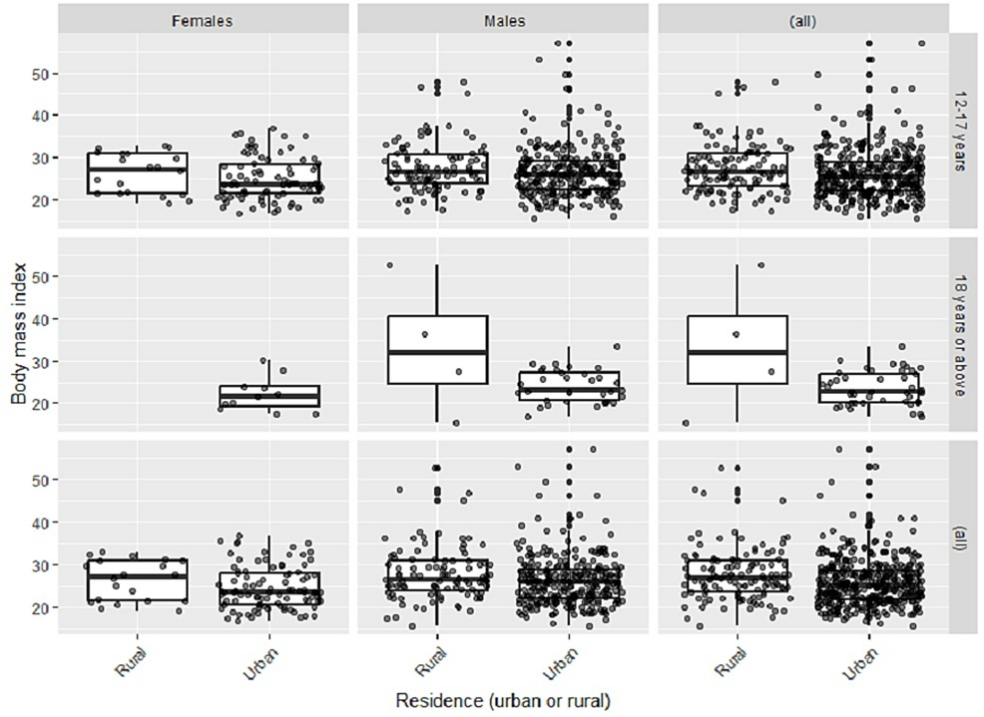
Body mass index of the studied population across different age groups, genders, and residences.

Figure 1 shows the distribution of the studied population based on their residence (urban vs. rural), gender, (male vs. females), and age category (12-17 years vs. 18 years or above).

Of the study participants, 33.6% were found to be overweight, while 20.5% were classified as obese. The prevalence of both obesity and overweight was notably higher in males than in females, with 20.9% of males being obese compared to 19.1% of females, and 36.5% of males being overweight compared to 23.5% of females. The age distribution appeared to significantly differ among the three weight groups (p = 0.001), with the majority of the overweight and obese individuals falling within the ≥18-year age group, while the normal group had a higher representation of individuals aged 12-17 years. Gender also showed a significant association with weight categories (p = 0.024), with a higher proportion of females in the normal group and more males in the overweight and obese groups. There were significant differences in the place of delivery among weight categories (p = 0.010), indicating potential regional variations in obesity rates. Mother’s education level exhibited significant variability among weight categories (p = 0.020), suggesting a possible link between maternal education and obesity. Social stress appeared to have a significant association with weight categories (p = 0.012), potentially influencing weight status. Physical activity levels showed a significant relationship with weight categories (p = 0.005), with more physically active individuals being less prevalent in the obese group (Table 2).

**Table 2:** Association between different modifiable and non-modifiable risk factors with weight gain. P-values <0.05 are significant. SAR = Saudi Arabian Riyal

Variables		Normal		Overweight		Obese		Test statistics	P-value
		Number	Percentage	Number	Percentage	Number	Percentage		
Age	12–17 years	26	53.1	23	46.9	0	0.0	14.59	<0.001
	≥18 years	209	45.1	149	32.2	105	22.7		
Gender	Female	66	57.4	27	23.5	22	19.1	8.92	0.012
	Male	169	42.6	145	36.5	83	20.9		
Residence	Rural	41	34.5	45	37.8	33	27.7	9.19	0.010
	Urban	191	49.4	127	32.3	72	18.3		
Place of delivery	Abha	105	49.3	68	31.9	40	18.8	14.60	0.024
	Kamis Mushayt	39	48.1	30	37.0	12	14.8		
	Tanoma	8	20.0	18	45.0	14	35.0		
	An-Namas	83	46.6	56	31.5	39	21.9		
Living condition	Alone	21	45.7	19	41.3	6	13.0	2.27	0.322
	Family	214	45.9	153	32.8	99	21.2		
Family size	Below 6	64	41.0	57	36.5	35	22.4	7.21	0.125
	Between 6 and 8	123	45.6	87	32.2	60	22.2		
	Above 8	48	55.8	28	32.6	10	11.6		
Family income	Less than 5,000 SAR	15	68.2	5	22.7	2	9.1	10.93	0.091
	5,000–10,000 SAR	43	50.0	24	27.9	19	22.1		
	10,000–20,000 SAR	103	42.7	94	39.0	44	18.3		
	Above 20,000 SAR	74	45.4	49	30.1	40	24.5		
Parental marital status	Married	217	46.0	159	33.7	96	20.3	0.11	0.948
	Single	18	45.0	13	32.5	9	22.5		
Mother’s education	Illiterate	26	42.6	17	27.9	18	29.5	21.10	0.020
	Postgraduate	20	58.8	7	20.6	7	20.6		
	Preparatory	20	60.6	9	27.3	4	12.1		
	Primary education	28	52.8	14	26.4	11	20.8		
	Secondary	35	34.0	50	48.5	18	17.5		
	University	106	46.5	75	32.9	47	20.6		
Father’s education	Illiterate	9	60.0	3	20.0	3	20.0	10.60	0.389
	Postgraduate	24	41.4	21	36.2	13	22.4		
	Preparatory	20	50.0	13	32.5	7	17.5		
	Primary	15	35.7	16	38.1	11	26.2		
	Secondary	63	54.8	37	32.2	15	13.0		
	University	104	43.0	82	33.9	56	23.1		
Smoking	Ex-smoker	12	36.4	11	33.3	10	30.3	8.43	0.077
	Non-smoker	179	44.9	133	33.3	87	21.8		
	Smoker	44	55.0	28	35.0	8	10.0		
Social stress	No	178	48.0	129	34.8	64	17.3	8.80	0.012
	Yes	57	40.4	43	30.5	41	29.1		
Watching television	No	62	44.3	56	40.0	22	15.7	7.29	0.698
	Yes	173	46.5	116	31.2	83	22.3		
Sleeping hours	Less than 6 hours	62	43.7	47	33.1	33	23.2	3.14	0.535
	Between 6 and 9 hours	150	46.2	108	33.2	67	20.6		
	More than 9 hours	23	51.1	17	37.8	5	11.1		
Watching smartphones	Less than an hour per day	13	35.1	13	35.1	11	29.7	7.10	0.312
	Between 1 and 2 hours	32	40.5	27	34.2	20	25.3		
	I don’t	35	54.7	21	32.8	8	12.5		
Physical activity	Less than 3 times per week	104	47.5	69	31.5	46	21.0	14.84	0.005
	More than 3 times a week	78	54.5	47	32.9	18	12.6		
	I do not	53	35.3	56	37.3	41	27.3		

Table 3 presents the results of an ordinal logistic regression analysis, showing the odds ratios (ORs) and their corresponding 95% confidence intervals (95% CIs) for various predictor variables in relation to the reference category. The OR represents the change in the odds of being in a higher weight category (overweight or obese) compared to the reference category (normal). The CI provides a range within which the true population OR is likely to fall. Females had 2.31 (95% CI = 1.45-3.71) times higher odds of being in a higher weight category (overweight or obese) compared to males. Individuals aged 12-17 years had 0.53 (95% CI = 0.28-0.97) times lower odds of being in a higher weight category compared to individuals aged 18 years or older. Individuals born in Tanoma had 2.32 (95% CI = 1.13-4.75) times higher odds of being in a higher weight category. Individuals belonging to larger families (with more than eight members) had 0.43 (95% CI = 0.24-0.74) times lower odds of being in a higher weight category compared to families with fewer members. Individuals from families with an income of above 20,000 SAR had 3.79 (95% CI = 1.38-11.35) times higher odds of being in a higher weight category. Respondents who smoked had 0.26 (95% CI = 1.31-2.93) times lower odds of being in a higher weight category compared to non-smokers. Adolescents who experienced social stress had 1.96 (95% CI = 1.96-2.93) times higher odds of being in a higher weight category. Individuals who engaged in physical activity less than three times a week had 0.49 (95% CI = 0.32-0.75) times lower odds of being in a higher weight category compared to those who engaged in physical activity more frequently. Individuals who engaged in physical activity more than three times a week had 0.36 (95% CI = 0.22-0.58) times lower odds of being in a higher weight category compared to those who engaged in physical activity less frequently.

**Table 3:** Factors influencing weight status: results from the multinomial logistic regression analysis. P-values <0.05 are significant. SAR = Saudi Arabian Riyal

Variables	Odds ratio	Lower confidence interval	Upper confidence interval
Gender (Female)	2.31	1.45	3.71
Age (12–17 years)	0.53	0.28	0.97
Mother’s education (Primary)	0.54	0.24	1.20
Mother’s education (Preparatory)	0.48	0.18	1.25
Mother’s education (Secondary)	1.03	0.48	2.24
Mother’s education (Postgraduate)	0.47	0.17	1.28
Mother’s education (University)	0.66	0.32	1.37
Father’s education (Primary)	3.23	0.88	12.88
Father’s education (Preparatory)	2.33	0.59	9.89
Father’s education (Secondary)	2.14	0.58	8.66
Father’s education (University)	3.11	0.85	12.42
Father’s education (Postgraduate)	2.67	0.68	11.33
Place of delivery (Khamis Mushayt)	0.95	0.57	1.60
Place of delivery (Tanoma)	2.32	1.13	4.75
Place of delivery (Other)	1.11	0.73	1.67
Family size (6–8 individuals)	0.81	0.54	1.22
Family size (Above 8 individuals)	0.43	0.24	0.74
Family income (5,000–10,000 SAR)	2.62	0.95	7.84
Family income (10,000–20,000 SAR)	2.25	0.86	6.50
Family income (above 20,000 SAR)	3.79	1.38	11.35
Smoking (Non-smoker)	0.73	0.36	1.49
Smoking (Smoker)	0.26	0.11	0.59
Social stress (Yes)	1.96	1.31	2.93
Watching Television (No)	0.95	0.64	1.41
Physical activity (Less than three times a week)	0.49	0.32	0.75
Physical activity (More than three times a week)	0.36	0.22	0.58
Model parameters: Residual deviance: 984.3302, AIC: 1040.33			

## Discussion

This survey was designed to assess the prevalence of obesity and overweight among adolescents in the Aseer region of Saudi Arabia. Additionally, it aimed to highlight the factors that contribute to these conditions. Study participants were recruited using a multistage random sampling technique. The study analyzed the relationships between various independent variables and the likelihood of being in different weight categories (normal weight, overweight, and obese) using ordinal logistic regression. The results revealed several noteworthy findings. Females were less likely to be overweight compared to males. Individuals residing in rural areas had higher odds of being overweight or obese. Family sizes of below six members and between six and eight members were associated with an increased probability of obesity compared to larger family sizes. High family income was associated with higher odds of being overweight or obese, while no significant association was found for the low- and middle-income range. Smoking reduced the odds of being overweight or obese compared to non-smoking. The presence of social stress was associated with higher odds of being overweight or obese. Moreover, physical inactivity was linked to higher odds of being overweight or obese compared to engaging in physical activity more than three times a week.

The overall prevalence of obesity and overweight among the studied adolescents was 54.1% (20.5% for obesity and 33.6% for overweight). This figure is higher than that reported by El Mouzan et al. [22], who reported an overall prevalence of overweight, obesity, and severe obesity in all age groups at 23.1%, 9.3%, and 2%, respectively. Al-Hussaini et al. [23] reported an overall prevalence of overweight and obesity at 13.4% and 18.2%, respectively. In this study, a significant association was noted between the male sex and both obesity and overweight in bivariate and multivariate analysis. This finding has been consistently addressed in Saudi Arabia [24]. On the other hand, females were found to be obese more often than males based on the findings of Al-Hussaini et al. [23]. The prevalence of overweight was higher in girls (14.2%) compared to boys (12%), and this difference was statistically significant with a p-value of 0.02. This observed difference may be due to the different study settings and habits of participants. For example, Al-Hazzaa et al. [24] explained the higher rate of obesity among adolescent females as they were more sedentary and less active compared to males. Given the diversity in study settings and participant habits across different studies, conducting additional investigations becomes essential to gain a deeper understanding of the factors contributing to gender disparities in overweight and obesity.

In fact, parental characteristics play a crucial role in shaping children’s health behaviors and overall development. Previous research has suggested that maternal characteristics may have a more significant impact on children’s health status compared to paternal characteristics [25,26]. In this study, we observed a significant association between maternal education and adolescents’ weight in bivariate analysis; however, this association was not observed in multivariate analysis. Paternal educational level was not significant in either bivariate or multivariate analysis. Similarly, Feng et al. [27] reported that children whose mothers had a higher education level were more likely to be overweight or obese. Moreover, Yang et al. [28] indicated that the level of education among mothers influenced their children’s weight status. On the other hand, numerous studies conducted in Western countries have consistently shown a negative correlation between the educational level of mothers and the prevalence of overweight and obesity among children [29,30].

We found that smokers had a lower chance of being overweight or obese. Likewise, Jacobs [31] demonstrated a negative association between cigarette smoking and BMI for both males and females. It was observed that people who smoke more have lower BMI compared to infrequent or non-smokers. This inverse relationship between smoking behavior and BMI suggests that smoking may have an impact on body weight regulation. However, it is essential to note that while smoking may be associated with lower BMI, it poses significant health risks and should not be considered a healthy or recommended method for weight management. The adverse health consequences of smoking far outweigh any potential benefits related to weight control. Smoking cessation and the adoption of healthier lifestyle choices remain critical to overall well-being and weight management. Furthermore, other studies reported that smoking can increase weight [32,33].

In this study, social stress was associated with an increased probability of being overweight and obese. Prolonged elevation of cortisol levels due to chronic stress can have several adverse effects on the body. One consequence is an increase in appetite, which can lead to overeating and weight gain. Furthermore, chronic stress can lead to insulin hypersecretion, leading to insulin resistance and potentially contributing to the accumulation of (visceral) fat in the long term [34]. Additionally, chronic stress has been associated with various changes in adipose tissue, such as adipocyte hypertrophy, the promotion of preadipocyte conversion to mature adipocytes, and the activation of stromal fat immune cells [35]. Furthermore, chronic stress has been associated with the development of non-alcoholic fatty liver disease, which can result from the induction of oxidative stress and inflammation [36].

Sedentary behavior is recognized as an independent risk factor for chronic diseases and is responsible for approximately one in six deaths. This group of behaviors, marked by minimal energy expenditure, is widely prevalent in modern society. Even if an adolescent plays sports for an hour three times a week, they can still lead a relatively sedentary lifestyle compared to their peers from 50 years ago [37]. In this study, physical inactivity was a strong determinant of overweight and obesity. The rapid advancement of technology, which often leads to increased sedentary behavior, further complicates the challenges of physical inactivity.

The use of apps and virtual training partners can be a novel way to engage and motivate adolescents to be more physically active. Wearable technology integrated into smartphones, watches, and tablets can also play a significant role in monitoring physical activity levels and tracking biometric data, providing valuable insights and feedback to individuals about their activity patterns. Engaging the community is another significant strategy to enhance responses and outcomes for the initiatives implemented by health authorities. Community engagement has demonstrated its effectiveness in tackling various health challenges, including diabetes and obesity [38].

Strengths and limitations

The findings of our study should be considered in the context of its strengths and limitations. It is essential to acknowledge certain limitations of our study. The potential for recall bias in reporting the frequency of physical activity, sedentary behaviors, and dietary habits cannot be entirely ruled out. Moreover, being a cross-sectional study, we cannot infer causality from the findings, and further research with longitudinal designs would be necessary to explore causal relationships. Finally, the male sex and older adolescents’ predominance among participants may introduce selection bias. Despite these limitations, the robust sample size, random sampling technique, and inclusion of both sexes strengthen the validity of our study’s conclusions.

## Conclusions

The study’s main findings reveal that the overall prevalence of overweight and obesity in the study population is a cause for concern. Gender differences showed that being female was significantly associated with lower odds of being overweight or obese. Family size and income level also influenced adolescents’ weight. Furthermore, smoking and physical inactivity were linked to lower odds of being overweight or obese. These findings emphasize the importance of addressing demographic, socioeconomic, and lifestyle factors in combating childhood and adolescent obesity through targeted interventions.
